# Smoking Cessation Quitlines in Europe: Matching Services to Callers' Characteristics

**DOI:** 10.1186/1471-2458-10-770

**Published:** 2010-12-18

**Authors:** Marc C Willemsen, Regina M van der Meer, Gerard M Schippers

**Affiliations:** 1 STIVORO, Dutch Expert Centre on Tobacco Control, The Hague, The Netherlands; 2 CAPHRI, Maastricht University, the Netherlands; 3 Amsterdam Institute for Addiction Research, Academic Medical Centre, Amsterdam, The Netherlands

## Abstract

**Background:**

Telephone quitlines offer a wide range of services to callers, including advice and counsel, and information on pharmacotherapy for smoking cessation. But, little is known about what specific quitline services are offered to smokers and whether these services are appropriately matched to characteristics of smokers. This study examines how quitline services are matched to callers' level of addiction, educational level, stage-of-change with quitting, and whether they are referred by a doctor or other health professional.

**Methods:**

Between February 2005 and April 2006, 3,585 callers to seven European quitlines responded to our survey. During the course of and immediately after the call, quitline counsellors collected descriptive data on callers' characteristics and the services they used. We then conducted four logistic regression analyses to examine the relationship between quitline services and the four caller characteristics.

**Results:**

Forty three percent of all callers received information on pharmacotherapy - most often nicotine patches and nicotine gum - from the counsellor. As we predicted, these callers were the heavy smokers. There was a direct correlation between the length of the conversations between the counsellor and the educational level of the smoker: the lower the education of the smoker, the shorter the call. However, we found no significant association between any other type of service and the educational level of caller. We also found a correlation between the smoker's stage of quitting and the type of advice a counsellor gives. Smokers in the action stage of quitting were more likely to receive advice (in two quitlines) or counselling (in two quitlines) than those in the preparation stage, who were less likely to be referred (in three quitlines). Very few of the total number of calls (10.7%) were from referrals by health professionals. Referred callers were more likely to receive counselling, but this was found only in four of seven quitlines.

**Conclusion:**

Most of the services quitlines offer to smokers favour heavy smokers and those at a more advanced stage of cessation, but not based on their educational level. Thus, we recommend that European quitlines extend and tailor their services to include less-educated smokers.

## Background

Quitlines have become an important component of tobacco control efforts. Because of the demonstrated efficacy and the convenience of telephone-based counselling, there has been a rapid adoption of quitlines in Europe and worldwide since the 1990 s. However, facts about how quitlines operate is scarce.

There are a variety of ways in which counsellors respond to quitline clients [1]. First-time callers may receive a variety of services. These include self-help materials, pharmacological consultation, brief advice, and extensive counselling. Quitlines may also refer callers to health professionals for further information or for a medical prescription. Counsellors may refer smokers to smoking cessation clinics and proactive call-back services.

The manner in which counsellors respond to callers is important. Motivational interviewing is crucial to the success of telephone counselling. This patient-centred approach improves the efficacy of quitline counsellors [2] and is a common element in the training of counsellors in Europe. In a previous study we examined the effectiveness of nine quitlines [3]. We found that the type of behavioural support (counselling, brief advice, information) did not affect success rates. However, when a caller received additional telephone support, specifically through more telephone counselling, there was an increase in continuous abstinence. This finding was supported in a recent meta-analysis of the efficacy of different types of support provided by quitlines [4]. This review concluded that the number of calls may improve success rates, but failed to show that any particular method of counselling or adjunctive self-help materials leads to higher success rates. Regardless, according to clinical guidelines, pharmacotherapy should be advised to nicotine addicted smokers [5,6].

In this paper we examine whether the types of support quitlines offer is related to smokers' characteristics. Four important distinctions of smokers' characteristics were considered: level of addiction, education, caller's stage of change, and type of referral.

During the initial contact between the counsellor and the caller, the caller's level of addiction must first be assessed, which will determine whether or not information about pharmacotherapy is provided. A second consideration is educational level, since smokers with a lower education have more difficulty quitting [7,8]. The third smoker characteristic considered is the caller's stage of change. Quitlines cater to two groups of callers: smokers seeking help with quitting in the near future (those in the preparation stage of change - planning to quit within four weeks), and those callers who have already quit smoking but are having difficulty staying smoke-free (those in the action stage who may have quit for less than 6 months). The needs of callers in these two stages of smoking cessation are different from each other. In the preparation stage, smokers actively seek information on how to quit. In the action stage, smokers who are already trying to quit need continued support to reinforce their decision [9]. We hypothesised that compared to callers in the preparation stage, those in the action stage are more likely to get immediate attention. This group will tend to receive more direct counselling or advice from the quitline and fewer referrals to outside help or literature, regardless of whether they were self-referred or referred by a health professional.

We conducted this study by collecting data on all callers seeking help with quitting smoking. This included both those smokers without referrals and those referred to the quitline by health professionals - general practitioners, medical doctors, nurses, midwives, pharmacists. Thus, we were able to categorize the callers based on referrals and how referred callers differed from those without referrals. We anticipated that referred callers would more often receive counselling, since many health professionals consider smoking cessation counselling as too time consuming to do it themselves [10-12].

## Methods

### Data

The first European quitline began in 1988 and the number of quitlines has now grown to 30. In 2002, when we began recruiting quitlines for our research, the number had grown to 15. All quitlines are part of the European Network of Quitlines (ENQ), and each runs independently and is at a different stage of development. We tried to include a wide range of quitlines, from different parts of Europe, and excluded quitlines without the capacity to recruit sufficient respondents. The seven quitlines participating in this study were from Denmark, France, Ireland, Italy, the Netherlands, Norway, and the United Kingdom. Initially, German and Portuguese quitlines were also included but were dropped because they were unable to recruit enough callers during the period of the study.

### Respondents and procedure

From February 2005 to April 2006, counsellors in each of the seven quitlines screened all the calls for eligibility. All callers requesting telephone support to stop smoking were asked if they were in the preparation stage (intending to quit smoking within the next four weeks), or the action stage (had quit smoking in the last six months and were calling to prevent relapse). Those who were in the preparation or action stage were eligible for our study and were asked for informed consent. Those who called for other reasons, such as looking for information or advice on cessation methods but not yet ready to quit, or calling for another person, were excluded. The counsellors collected data and recorded it on a paper-and-pencil questionnaire (see additional file 1) at three points in the interview: during the call, while the caller was still on the line but the reason for the call had ended, and after the call had ended. At each quitline, data collection ended when 500 callers had been enrolled, or at the end of the inclusion period of 15 months, whichever came first.

### Questionnaire

In our questionnaire, the phrase *Type of Service *referred to one or more of the following items:

*• Literature sent was referred to as booklets or leaflets on quitting*.

*• Basic information *was a quick call providing objective or neutral facts about the consequences of stopping smoking.

*• Advice *was recommendations for how to quit smoking.

*• Pharmacotherapy information *was objective or neutral information about pharmacotherapeutic aids for smoking cessation.

*• Referral to a quit smoking service *provided by an outside agency, such as a stop smoking group.

*• Referral to a health professional *included a general practitioner, medical doctor, nurse, midwife, pharmacy, dentist, or other categories.

*• Counselling *was a caller-centred, person-tailored, in-depth motivational interaction.

The length of each call was recorded in minutes and excluded the time required to complete the questionnaire. Education level was the highest level obtained by the respondent. Exact definitions varied per quitline and depended on classifications that were common in each country. Quitline representatives categorized the answers so that for each quitline we obtained roughly comparable education categories (low, medium, high). We used the Heaviness of Smoking Index (HSI) to measure nicotine dependence. This index measures heaviness of smoking by combining two variables: the time to the first cigarette of the day and number of cigarettes smoked per day [13,14]. Heavy smokers have a score of 4 or greater on a scale ranging from 0 to 6. Counsellors asked callers if someone referred them to the quitline, and if yes, by whom, a health professional (a general practitioner, medical doctor, nurse, midwife, pharmacy, or a dentist) or other. If the response was no, callers were asked if they were self referred.

### Statistical analyses

Logistic regression analyses were conducted to identify those services (independent variables) that predicted the dependent variables (callers' characteristics). Four separate analyses were conducted, each with one of the following dependent variables: referral by a health professional (Yes = 1; No = 0), being heavy smoker (1) or a lighter smoker (0), low education (1) or a medium to high education (0), and being in the action stage of quitting (1) or in the preparation stage (0). To determine whether the association between type of service and caller characteristics might be modified by quitline, we examined the significance of the Quitline × Type of service interaction. If any interactions were significant, analyses were stratified by quitline or groups of quitlines. In all analyses, quitline was entered as a covariate to correct for between-quitline variation, and the service types were entered in the analysis simultaneously, to correct for overlap in services (method = enter). All analyses were performed using SPSS version 16.0 for Windows.

## Results

### Callers' characteristics

8,761 callers from seven European quitlines were checked for eligibility. Of these, 3,585 (40.9%) participated in the study. Main reasons for non-enrollment were not being in either the preparation or the action stage of smoking, or not calling for themselves. In addition, 12.7 percent did not give informed consent to participate in the study, and some callers met the eligibility criteria but were not recruited because they were distressed or abusive callers (4.9%), they had a language barrier (1.2%), or the counsellor forgot to ask the caller to participate (3.1%), or did not ask to participate (12.0%).

Table 1 presents the characteristics of the callers. Almost two thirds of respondents were female (63.4%). The mean age was 43.3 years (SD = 13.2). The distribution of educational level was: 29.3 percent low, 40.7 percent medium, and 30.0 percent high. Of all respondents, 31.9 percent were in the action stage and 68.1 percent were in the preparation stage. Smokers in the preparation stage were asked if they had set a quit date and 41.2 percent said they had. 48.3 percent were heavy smokers and more than 91 percent smoked at least 10 cigarettes per day. The mean number of cigarettes per day was 21.6 (SD = 11.7).

**Table 1:** Caller characteristics per quitline

	DM	FR	IR	IT	NL	NO	UK	Total
Number of respondents	425	619	494	520	493	534	500	3,585
Female (%)	65.8	61.1	55.3	57.3	73.4	73.0	58.5	63.4
Age (mean, SD)	46.2 (14.1)	42.0 (12.5)	40.3 (12.5)	42.3 (12.1)	46.0 (13.3)	44.5 (13.8)	42.2 (13.3)	43.3 (13.2)
Educational level (%)								
Low	26.1	35.9	22.1	33.2	27.9	20.8	38.5	29.3
Medium	49.8	23.8	39.9	51.5	44.2	43.2	36.4	40.7
High	24.1	40.2	38.0	15.3	27.9	36.0	25.1	30.0
Smokes >= 10 cigarettes per day (%)	91.7	91.9	89.4	94.8	92.7	86.9	92.4	91.4
Heaviness of Smoking Index score >= 4) (%)	55.5	46.4	48.5	46.3	58.0	39.3	46.3	48.3
Action stage of quitting (% yes)	32.0	33.0	38.5	11.3	31.4	41.4	35.8	31.9
Did someone refer you to our quitline?(%)								
Health Professional	14.1	13.6	6.1	3.8	14.0	11.6	12.0	10.7
Self-referral	75.8	68.7	76.5	81.2	75.7	63.1	66.0	72.2
Family/friends/colleagues	5.9	9.4	12.8	9.8	6.1	17.6	7.0	9.9
Other	4.2	8.4	4.7	5.2	4.3	7.7	15.0	7.2

Note. DM = Danish quitline, FR = French quitline, IR = Irish quitline, IT = Italian quitline, NL = Dutch quitline, NO = Norwegian quitline, UK = United Kingdom quitline.

### Types of services

For 82.9% of respondents, the call being studied was the subjects' first to a quitline. The mean length of calls was 15.3 minutes (SD = 9.8), with only 21.3% of calls lasting longer then 20 minutes. Most calls resulted in combinations of various services (see Table 2). Counselling was the most frequent type of service provided (76.3%) and information about pharmacotherapy was received by 43.6%. Of this group, 19.4% of information was about bupropion, 83.6% about nicotine patches, 51.2% about nicotine gum, 12.2% about lozenges, 8.5% about inhalers, and 6.0% about sub-lingual tablets. In some cases, information about more than one pharmacotherapy was given that resulted in an overlap in percentages.

**Table 2:** Types of services provided to callers per quitline

	DM	FR	IR	IT	NL	NO	UK	Total
Service provided (%)^a^								
Literature sent ^b^	7.1	51.9	78.3	31.7	-	71.0	60.0	44.1
Basic information (quick call)	76.7	66.1	72.9	45.6	55.0	83.7	43.2	63.2
Advice on how to quit	27.1	71.6	88.7	37.3	2.0	81.3	35.2	50.5
Pharmacotherapy information	48.0	74.0	51.0	9.4	32.5	51.5	33.0	43.6
Referral to outside service (such as a smoking cessation course)	10.8	1.1	4.3	62.3	8.7	5.8	27.2	17.0
Referral to a health professional	4.0	14.4	14.8	16.5	3.9	16.9	13.4	12.3
Counselling	66.1	87.4	91.5	60.0	51.9	94.8	77.8	76.3
Mean length of call (minutes; mean, SD) ^c^	18.7 (11.1)	19.6 (11.9)	16.4 (8.4)	10.0 (5.8)	10.0 (4.5)	18.7 (10.4)	12.9 (7.5)	15.3 (9.8)

Note. DM = Danish quitline, FR = French quitline, IR = Irish quitline, IT = Italian quitline, NL = Dutch quitline, NO = Norwegian quitline, UK = United Kingdom quitline.

^a ^Multiple answers allowed. ^b^Data for the Netherlands missing ^c ^N = 3,337

### Association between type of service and caller characteristics

Logistic regression analysis showed that heavier smokers were more likely to receive information about pharmacotherapy than lighter smokers (see Table 3). In addition, in comparison to lighter smokers, heavy smokers were more likely to be referred to a health professional and to receive counselling. These results were not modified by the specific quitline (no significant interaction), suggesting a consistent finding across quitlines.

**Table 3:** Association between callers' heaviness of smoking (0 = HSI score <4, 1 = HSI >= 4) and types of quitline service (Results of logistic regression analyses - OR).

Type of service	OR (95% CI)
Literature sent	.89 (.75 - 1.04)
Basic information (quick call)	.94 (.81 - 1.09)
Advice on how to quit	1.03 (.87 - 1.22)
Pharmacotherapy information	1.30 (1.12 - 1.51) ***
Referral to outside service (eg., stop smoking course)	1.15 (.93 - 1.43)
Referral to a health professional	1.35 (1.02 - 1.54) *
Counselling	.92 (.78 - 1.09)

*p < .05,** p < .01, *** p < .001; NB. Quitline included as co-variate in analysis.

With respect to education level, the findings were not consistent across quitlines. There were significant interactions (all p < .05) between Quitline and Information about pharmacotherapy, Quitline × Referral to a health professional and Quitline × Counselling. The results were therefore stratified by quitline (see Table 4). However, in none of the seven quitlines, callers with low education were more likely to be counselled. The only two significant findings were that in the Dutch quitline low educated callers were more likely to receive information on pharmacotherapy (OR = 2.27; 95% CI = 1.46-3.51) while counsellors in the UK quitline were more likely to refer low educated smokers to a health professional (most likely community smoking cessation services) (OR = 2.68; 95% CI = 1.40-5.12). An additional finding was that, on average, the duration of the call was longer with more highly educated callers (17.11 minutes) than with callers who had a medium (14.72 minutes) or a low (14.38 minutes) education (F = 22.45; df = 2; p < .001).

**Table 4:** Association between educational level (1 = Low, 0 = Medium or High) and types of quitline service (Results of logistic regression analyses -OR - per quitline)

	DM	FR	IR	IT	NL	NO	UK
Number of respondents	410	604	426	520	491	528	390
Type of service							
Literature sent	.84	1.10	1.02	1.01	-	1.14	1.12
Basic information (quick call)	.94	1.16	1.30	1.19	1.04	.74	1.65
Advice on how to quit	.92	.81	.64	1.49	1.25	1.34	.96
Pharmacotherapy information	1.04	.87	.92	1.29	2.27***	.97	.94
Referral to outside service (eg., stop smoking course)	1.17	.27	.46	.87	1.29	.56	.84
Referral to a health professional	1.27	1.54	1.19	.65	1.43	1.12	2.68***
Counselling	.80	1.51	.77	.44	.77	1.05	1.22

Note. DM = Danish quitline, FR = French quitline, IR = Irish quitline, IT = Italian quitline, NL = Dutch quitline, NO = Norwegian quitline, UK = United Kingdom quitline.

*** p < .001, ^# ^number of respondents lower compared to table 1 due to missings on the education variable

The relationship between services and stage of change was modified by quitline location, as indicated by significant interactions between quitline and most types of services. Again, the results had to be stratified by quitline (Table 5). In the Danish, Italian, and English quitlines, respondents in the action stage were referred to outside help less often. In addition, respondents in the Irish quitline were less likely to be referred to a health professional in the action stage than in the preparation stage. Respondents in the action stage were more than twice as likely to receive advice on how to quit than respondents in the preparation stage, but this was only significant for the French and Irish quitlines. The Italian and Dutch quitlines were significantly more likely to give counselling to respondents in the action stage of change.

**Table 5:** Association between stage of change (1 = Action stage, 0 = Preparation stage) and types of quitline service (Results of logistic regression analyses - OR - per quitline)

	DM	FR	IR	IT	NL	NO	UK
Type of service							
Literature sent	.44	.73	.77	1.28	-	.32***	.58**
Basic information (quick call)	.65	.80	.70	.45*	1.04	1.18	.95
Advice on how to quit	.78	2.16***	2.19*	.99	1.34	.90	1.09
Pharmacotherapy information	.70	1.01	.69	.53	.76	1.01	.90
Referral to outside service (eg., stop smoking course)	.42*	.88	.35	.15***	.51	.50	.19***
Referral to a health professional	.23	.86	.27***	.53	1.51	1.26	.63
Counseling	1.42	1.28	.88	2.79*	1.58*	.50	1.09

Note DM = Danish quitline, FR = French quitline, IR = Irish quitline, IT = Italian quitline, NL = Dutch quitline, NO = Norwegian quitline, UK = United Kingdom quitline.

*p < .05,** p < .01, *** p < .001;

Most calls to quitlines (72.2%) were self-referred. Only 10.7% had been referred by a health professional; the rest were referred by family, friends or others. A logistic regression analysis revealed that the association between being referred and type of services received was modified by quitline location. In the Danish, French, and UK quitlines we found a positive association with referral to a medical professional, but not in the other quitlines (Table 6). Instead, in these four quitlines we found a positive association between medical referral and receiving information about pharmacotherapy and being counselled.

**Table 6:** Association between being referred to the quitline (yes = 1, no = 0) by a health professional and types of quitline service

	OR (95% CI)	
Type of service	Danish, French, UK quitlines	Irish, Italian, Dutch, Norwegian quitlines
Literature sent	1.02 (.72 - 1.46)	.81 (.52 - 1.25)
Basic information (quick call)	.77 (.55 - 1.08)	1.35 (.93 - 1.94)
Advice on how to quit	.71 (.50 - 1.00)	.84 (.52 - 1.36)
Pharmacotherapy information	1.15 (.81 - 1.62)	1.51 (1.08 - 2.10) *
Referral to outside service (eg., stop smoking course)	1.48 (.92 - 2.39)	1.38 (.82 - 2.33)
Referral to a health professional	6.26 (4.28 - 9.16) ***	.89 (.54 - 1.47)
Counselling	1.10 (.71 - 1.44)	1.59 (1.04 - 2.45) *

*p < .05,** p < .01, *** p < .001; NB. Quitline included as co-variate in analysis.

## Discussion

For this study, we used data from seven European quitlines to examine which callers get which types of service from quitlines. Although some findings were consistent across quitlines, most seemed to be mediated by quitline characteristics.

As hypothesised, one consistent finding was that heavy smokers were more likely to receive information on pharmacotherapy, especially nicotine replacement therapy. This finding mirrors clinical guidelines on smoking cessation that recommend providing pharmacotherapy to more dependent smokers [5]. We also found that counsellors were likely to refer heavy smokers to a health professional, most likely to obtain a prescription for pharmacotherapy.

An unexpected finding was that low educated smokers, who generally have more difficulty quitting, were not getting intense counselling support. The length of call for lower educated smokers was shorter than for more highly educated smokers. This finding is disappointing since quitlines, with their centralized experience in smoking cessation, are particularly well placed to deal with disadvantaged groups [15]. When asked to explain these findings, quitline representatives justified the lengthier calls because it was their impression that highly educated smokers requested more specific and detailed guidance compared to the lower educated. Furthermore, it was their experience that higher educated callers are more willing to expressing fears and ask more questions, making it easier for them to be counseled. Therefore, we advise European quitlines to improve their counsellor training and support systems so that counsellors are better equipped to communicate with smokers from lower educated strata.

Smokers who had already quit and called for relapse prevention (action stage) were more likely to receive advice (in two quitlines) or counselling (in two quitlines) than smokers preparing to quit. They were also more likely to be further referred (in three quitlines). This finding partly confirmed our hypothesis that more acute crisis calls get more immediate attention from quitline counsellors. Further research should focus on why this finding was not the case in all quitlines.

This study also makes it clear that there are differences in how callers to EU quitlines are served. In particular, we found that callers referred to quitlines by health care providers were likely to receive counselling, although this association was significant in only four of seven quitlines. This finding suggests that quitlines can play a role in supporting physicians who seek additional counselling for their smoking patients. It also suggests that further research is needed to examine more closely the different practices of quitlines in different countries.

From a public health perspective, it appears that despite national differences in quitline services, not many patients seem to find their way to quitlines yet, since only 10.7% of calls were referrals from health care providers. In contrast, in California, between 14.5% and 51.3% of callers (variance depended on whether they used nicotine replacement therapy) heard about the quitline from a health care provider [16]. It seems worthwhile for European quitlines to put more effort into bridging the current gap between the health care system and quitlines. This could be done, for example, by experimenting with pro-active enrolment models where names and telephone numbers of patients are forwarded to the quitline. Such systems have been shown to increase the use of quitline services by referred patients [17].

Our study has both strengths and limitations. One strength is that some findings were consistent across quitlines. These findings will probably hold true for many quitlines in developed countries beyond those studied here. However, because there was so much variance among quitlines, it is difficult to make general conclusions without further examining how quitline or country variables may mediate results. The conceptual framework that we developed previously could be useful for guiding such future research [3]. In this framework, we identified the factors that are most likely to directly or indirectly affect the types of quitline services provided to callers. These factors were identified at the micro level (caller characteristics and counselor variables), the meso level (quitline organizational factors), and the macro level (mass media promotion, the health care system, and tobacco control environment, including differences in tobacco epidemic and restrictiveness and type of control measures).

In our research we found that how quitlines matched their services to caller characteristics varied. This raises an important question: Is more standardisation in quitline protocols needed to match services to caller characteristics? Further research might identify the most effective caller/service combinations. In particular, qualitative research is needed to determine which individual quitline protocols can be successfully adapted to local settings, so that other quitlines can learn from these best practices. An important task for the international quitline networks is to disseminate this information among network members.

One limitation of the study is that we did not ask callers their preferences for type of service. Therefore, we do not know to what extent the type of service rendered is the result of a specific request from the caller. Counsellors are expected to match the services offered to the needs of the caller. This requires following formalized protocol. For instance, counsellors are advised to follow ENQ quitline protocols, rather than giving callers what they ask for. However, it is unclear how smokers' preferences influence counsellors' decisions. There is consensus among smoking cessation experts that evidence, patient preference, and patient experience are important considerations when deciding the best treatment [18]. Future quitline research should take into account caller characteristics as well as callers' needs, variations in quitline protocol, counsellor characteristics such as level of experience and training, and the quitline's level of maturity.

This study was limited to one-session calls. We do not know whether calls resulted in further call-back appointments or whether the advice callers received from counsellors were followed. Future studies should monitor the callers' actions after their initial call.

Another potential limitation of this study is that we cannot be sure how accurately counsellors' recorded callers responses to our questionnaire and if it was done consistently across quitlines. We are confident, however, that this was not a problem because we used four strategies to increase classification reliability. First, representatives from all seven quitlines helped us to accurately define the services used in this study. Second, we conducted a full day's training workshop for representatives of all quitlines. This included how to implement the study protocol, as well as how to record callers' responses on the score sheet. Third, we provided detailed written instructions to all quitlines that could be used to train their own counsellors in the research tasks. Finally, the definitions of the services were printed on the questionnaire in close proximity to the pertinent questions, so the counsellors always had the definitions at hand when they had to record the type of service they had provided. However, despite these precautions, some differences in interpretation may have occurred.

## Conclusion

The broadest conclusion we can make from this study's findings is that, in general, quitline services appropriately matched the smoker's level of addiction (as measured by the Heaviness of Smoking Index) and the smoker's stage of change. Our findings also provided evidence that quitlines might improve their services in two areas. First, we found that quitlines did not consider the educational level of callers in their responses. Therefore, we advise European quitlines to improve their services for this important target group. This is especially important in light of the increasing socio-economic gradient of the smoking problem in developed countries. Second, we found that there are a low number of referrals to quitline services by European health professionals. Based on this, we strongly advise quitlines and policy makers to explore ways of integrating quitlines into their country's health care system.

## Competing interests

The authors declare that they have no competing interests.

## Authors' contributions

MCW conceived of this study, drafted the manuscript, and performed the statistical analyses. RMM coordinated the data collection, participated in the design of the study, and helped draft the manuscript. GMS participated in the design of the study and helped to draft the manuscript. All authors read and approved the final manuscript.

## Pre-publication history

The pre-publication history for this paper can be accessed here:

http://www.biomedcentral.com/1471-2458/10/770/prepub

## Supplementary Material

Additional file 1 **Questionnaire**. This file contains the questionnaire that was used in the study. Click here for file
