# Supplementary material for: Smoking Cessation Quitlines in Europe: Matching Services to Callers' Characteristics
Source: BMC Public Health. 2010 Dec 18;10:770. doi: 10.1186/1471-2458-10-770 (PMC3020686; doi:10.1186/1471-2458-10-770)
Supplement: Additional file 1 — Questionnaire. This file contains the questionnaire that was used in the study. [file 1471-2458-10-770-S1.DOC]

**ESCHER questionnaire for callers** (version UK 12/1/2005)

**Fill in the questionnaire for every caller who calls for telephone support for smoking cessation (information, advice and/or counselling)**

ID:

Date: ….. / ….. / 05 (d/m/y)

-------------------------------------------------------------------------------------------------------------------

[part V at start of questionnaire, left hand side instead of right hand side]

**V**

**Caller’s details**

*We would like to call you again in a year and ask you some of the same questions. Therefore we need your address and telephone numbers.*

ID:

First name & Surname: ………………………………………………………………………..

Address: ………………………………………………………………………..

………………………………………………………………………..

Town/City: ………………………………………………………………………..

Post code: ………………………………………………………………………..

Email: …………………………………………………………………………

*May I have two phone numbers, because we would like to call you again in a year.*

*And therefore it is important that we can get in touch with you.*

Tel (home): ………………………………………………………………………..

Tel (work): …………………………………………………………………………

Tel (mobile): ………………………………………………………………………..

**(Copy from part IV question 18)**

Date of birth: …../……../…….. (d/m/y)

**Go to part VI**

**Make sure you fill in part VI immediately after finishing the call**

------------------------------------------------------------------------------------------------------------------

**I**

**Eligibility**

A caller is eligible for the recruitment when he/she meets one of the following two criteria (fill in):

2. He/she quits smoking in the last six months and calls for preventing relapse (action stage)

1. He/she wants to quit smoking within one month (preparation stage)

OR

O No O No Not eligible Fill in part III

O Yes O Yes

Eligible: Fill in question A Eligible: Fill in question B and C

A. Did he/she set a quit date? B. How long since he/she did quit smoking?

O No ……… days ago

O Yes, When? ……… weeks ago

……/……../…….. (d/m/y) …….. months ago

C. Has he/she smoked in the past 6 days

O No

O Yes

Go to part II Go to part II

**III**

**Not recruited because**

(Fill in)

O Did not meet inclusion criteria

O Already participating in project

O Distressed or abusive caller

O Language barrier

O Forgot to ask caller to participate

O No time to ask caller to participate

O Client hangs up before having a chance to ask him/her

O Eligible but caller declined

O Other: …………………………………………

…………………………………………………….

→ End of the questionnaire

→ Fill in part V if you want to send the caller some literature.

**II**

**Informed consent**

Use the following highlighted words to make your own text or literally use the following text:

*We are participating in a European project for improving the quitline services throughout Europe.*

*This means that we would like to ask you some additional questions. The first couple of questions will take only ten minutes and can be done right now. A second questionnaire will be conducted one year from now by a telephonic research centre and will also take about ten minutes of your time. All answers will be held strictly confidential. If there are any questions you feel uncomfortable with you don’t need to answer them. Would you be interested in participating in this project?*

O No → *Thank you for calling. If you have more questions or you need support you can always call us* → Fill in III

O Yes

Is this the first time you have been asked to answer this additional questionnaire?

O No → *It is not necessary to ask you the questions again, because you already answered them. Thank you for participating in the project* → Fill in III

O Yes → Go to IV

**IV**

**Questions**

*Thank you for participating. First I like to ask you a few questions about your smoking habit, then a few questions about the quitline.*

*Maybe some questions will look similar to some I already asked you before, but for the research it is important that I ask these questions again but maybe in a slightly different way*

Instructions:

- Read out instructions to the caller shown in speech box in italics

- Ask the questions literally.

- Do not name the answer categories.

- Only ask questions if you do not know the answer yet!

- A variation to a response or an additional response is only acceptable to questions marked

Additional response acceptable.

- Some questions are required to be asked in either the present or past sense. These are marked in square brackets [ ]. For callers in the action stage these questions have to be asked in the past sense.

1. [Do you/did you] smoke daily or occasionally?

O Daily

O Occasionally (=less than 1 cigarette per day)

2. What [do you/did you] smoke?

(Additional response acceptable.)

O Cigarettes

O Hand-rolled cigarettes

O Cigars

O Pipe

3. How many [do you/did you] smoke?

(Additional response acceptable; if necessary ask for an average number)

………. Cigarettes (number per day)

………. Hand-rolled cigarettes (number per day)

………. Cigars (number per day)

………. Pipe (number per day)

4. How soon after you wake up [do you/did you] smoke your first cigarette?

O Within 5 minutes

O 6 to 30 minutes

O 31 to 60 minutes

O After 60 minutes

5. Have you reduced the amount you smoke[d] within the last month [before your quitdate]?

O Yes

O No

O Don’t know

6. Since you started smoking daily, how many times have you successfully quit using tobacco for at least 24 hours?

…………….(number of quit attempts) (if zero attempts go to question 8)

7. Thinking about your longest quit attempt, for how long did you stop?

……………. O hours/O days/O weeks/O months/O years

8. Have you ever used treatments or health professionals to support you quitting?

For example NRT, Zyban, self-help materials, stop smoking groups, counselling or an advice from a health professional.

(mark all that apply)

O No

O Yes → What kind of treatments or health professionals?

O Medication → O Zyban / Bupropion

O NRT – patches

O NRT – gum

O NRT – nasal spray

O NRT – inhaler

O NRT – lozenges

O NRT – sub-lingual tablet

O Self-help materials (booklets, videos, tapes, websites)

O Stop smoking group

O Individual counselling

O Quitline

O Advice from → O Medical doctor

O General Practitioner

O Nurse

O Other: …………………………………………

O Allen Carr → O Book

→ O Course

O Acupuncture / Softlaser therapy

O Hypnotherapy

O Other (please state): ……………………………………………………..

9. What specifically triggered you to decide to stop smoking?

(Single most important one)

O A health problem I have at present

O Better for my health in general

O Smoking is becoming anti-social

O Smoking bans

O Cigarette pack warning

O Aesthetic and cosmetic reasons (smell, stained teeth)

O I stopped liking it

O I don’t like being addicted / I want to take control of my life

O Doctor said I should stop

O Family/friends wanted me to stop

O The price of cigarettes

O Pregnancy / Family planning

O Worried about the effect on my children

O Other (please state)………………………………………………………………………

10. At what age did you start smoking daily?

……………….(age)

11. On a scale from 1-10, with 1 being not at all confident, and 10 being extremely confident, how confident are you that you will be able to stop smoking completely this time?

| Not at all  confident | 1 | 2 | 3 | 4 | 5 | 6 | 7 | 8 | 9 | 10 | Extremely  confident |
| --- | --- | --- | --- | --- | --- | --- | --- | --- | --- | --- | --- |

12. On a scale from 1-10, with 1 being not at all important, and 10 being extremely important, how important is it for you to stop smoking completely this time?

| Not at all  important | 1 | 2 | 3 | 4 | 5 | 6 | 7 | 8 | 9 | 10 | Extremely  important |
| --- | --- | --- | --- | --- | --- | --- | --- | --- | --- | --- | --- |

13. Are there relatives or friends or colleagues who support you in your quit attempt?

O Yes

O No

14. Do you have a partner?

O No → Go to question 18

O Yes → Go to question 15

15. Does your partner support you in your quit attempt?

O No

O Yes

16. Is your partner a smoker?

O No, partner never smoked → Go to question 18

O No, partner is an ex-smoker → Go to question 18

O Yes → Go to question 17

17. Does your partner want to quit smoking?

O No, doesn’t want to quit

O Yes, wants to quit

18. Could I have your date of birth?

…../……../…….. (d/m/y)

Introduction:

*The following three questions are personal questions. The reason for asking these questions is that these topics are related to smoking.*

19. Have you ever had 2 weeks or more during which you felt sad, blue, or depressed or when you lost all interest or pleasure in things that you usually cared about or enjoyed?

O No → go to question 21

O Yes

20. Did you have such a period of two weeks or more within the last month?

O No

O Yes

21. What is the highest level of education you have completed?

(caller states actual educational level and counsellor categorizes)

O GCSE

O A-level

O NVQ’s

O graduate

O Post graduate

22. Is this your first call to the quitline?

O No ⭢ How many times did you call to the quitline in the past?

………..(number of times)

O Yes

23. Where did you see the quitline number?

(Additional response acceptable)

O Mass Media ⭢ O Radio

O TV

O Newspaper

O Magazine

O Other advertising ⭢ O Billboard / transport

O Phone book / Yellow book

O Leaflets

O Internet

O Cigarette pack warning

O Other: ………………………………………………………………………………

24. Did someone refer you to our quitline?

(Additional response acceptable)

O No

O Yes ⭢ who? ⭢ O Health professional ⭢ O General Practitioner O Medical doctor O Nurse

O Midwife

O Pharmacy

O Dentist

O Other health professional

…………………………………………

O Family / friends / colleagues

O Self-referral

O Other: …………………………………

25. Overall, how satisfied were you with the service you received from the quitline?

Is this very, mostly, somewhat or not at all satisfied?

O Very satisfied

O Mostly satisfied

O Somewhat satisfied

O Not at all satisfied

**Fill in part V – the caller’s details**

**After filling in part V:**

**Thank the caller for participating and finish the call**

Summarize the call with two lines about the counselling part and two lines about the European project.

For example:

*This was my last question. Thank you for participating in the project. You called us for……..*

*……………………… If you have more questions or you need more support give us a call.*

**Go to part VI**

**Make sure you fill in part VI immediately after finishing the call**

**VI**

**Output**

1. Sex: O Male

O Female

2. Outcome / Intervention (mark all that apply):

O Basic information

O Specific information → O Medication: O Zyban O NRT (patches)

O NRT (gum)

O NRT (spray)

O NRT (inhaler)

O NRT (lozenges)

O NRT (sublingual tablet)

O Referrals: O Stop smoking group

O Allen Carr (book)

O Allen Carr (course)

O Acupuncture

O Hypnotherapy

O Health professional → O General Practitioner

O Medical doctor O Nurse

O Midwife

O Pharmacy

O Dentist

O Other health professional:

………………………………………..

O Other: …………………………………………………………….......

O Advice: What kind of advice?:……………………………………………………………………………

O Counselling: O Proactive

O Reactive

O Literature sent: O Flyer advertising the email service

O Quit smoking without putting on weight

O The quit guide to stopping smoking

O Other:…………………………………………………………………………………………………………………………………………

Definitions:

Basic information Objective / neutral information to the caller about facts, consequences of stopping smoking, cravings etc. (quick call)

Specific information Objective / neutral information to the caller about cessation methods, referral to the local smoking cessation services or referral to health professional

Advice Caller receives recommendations on how to quit smoking. For example what would be the best method and a recommendation for seeing a health professional.

Counselling Caller centred and person tailored, in-depth, motivational interaction

Literature sent Booklets / leaflets on quitting

Length of call (fill in):

……. Minutes excluding ESCHER-questionnaire

……. Minutes including ESCHER-questionnaire
